# An overview of the presentation and management of vulvovaginal cancers at the NSIA-LUTH Cancer Centre

**DOI:** 10.3332/ecancer.2025.1935

**Published:** 2025-06-27

**Authors:** Chidiebere I Agbakwuru, Eben Aje, Mutiu Jimoh, Anthonia Sowunmi, Samson Ezekiel

**Affiliations:** 1 Department of Radiation and Clinical Oncology, NSIA-LUTH Cancer Center, Lagos, Nigeria; 2 Department of Radiation Oncology, Lakeshore Cancer Center, Lagos, Nigeria; 3 Department of Clinical Research, NSIA-LUTH Cancer Center, Lagos, Nigeria

**Keywords:** vulvovaginal cancer (VVC), clinical presentation, risk factors, treatment, Nigeria

## Abstract

**Background:**

Vulvovaginal cancer (VVC) are two rare malignancies in women which are less prevalent than other gynecological cancer.

**Aim:**

This study seeks to investigate the evaluation of the presentation and management of VVC cases at our institution, aiming to contribute to a deeper understanding of VVC within sub-Saharan Africa.

**Methods:**

This was a retrospective study of all VVC cases seen at the NSIA-LUTH Cancer Centre from May 2019 to June 2024. Data on clinical presentation and treatment modalities were collated and analysed using the Statistical Package for Social Science version 27.0.

**Results:**

Out of a total of 10,376 cancer cases reported during the study period, 0.6% (67 cases) were accounted as VVC, the median age at presentation was 54 years range: 32–88 years). Vaginal bleeding (41.8%), lump (41.8%) and vaginal swelling (32.8%) were the major presenting symptoms. Immunosuppression Human Immunodeficiency Virus, alcohol, multiple sexual partners and family history of cancer were the most identified risk factors. Histological examination identified squamous cell carcinoma as the predominant subtype (74.8%). Most cases were diagnosed at stage IV (52.2%). Treatment uptake was average, with 23.9% received surgery, 40.3% receiving chemotherapy and 56.7% undergoing radiotherapy.

**Conclusion:**

The incidence of VVC is increasing in Nigeria, particularly among young women. This study showed late presentation and advanced-stage cancer with vaginal bleeding, lump and vaginal swelling as the most presenting symptoms. It is imperative to increase awareness, which potentially facilitates early detection of the disease.

## Background

Vulvar cancer and vaginal cancer are two rare malignancies in women which are less prevalent than uterine cancer of the endometrium, ovary and cervix, but vaginal is more common than vulvar cancer in the United States [1]. They both account for almost 10% of all gynecologic cancers, with approximately 6,000 newly diagnosed cases and 5,000 deaths yearly in the United States [1]. It is most commonly diagnosed during the sixties and seventies (age 51–60 years, 36%; age 61–70 years, 28.6%) [2]. However, predominantly a disease for elderly women with an average age of 67 years, but it’s increasingly becoming common among younger women. The incidence of vulvar and vaginal cancer is significantly high in multiparous (90%) and postmenopausal (65%) women [2].

The vulvovaginal cancer (VVC) burden in Nigeria is compounded by limited awareness of the disease, cultural barriers and poor access to preventive and diagnostic services, including HPV vaccination and regular gynecological screening. In sub-Saharan Africa, where cervical cancer is predominant in the spectrum of gynecological malignancies, less attention is given to cancers of the vulva and vagina despite a rising incidence attributed to an increase in HPV prevalence [3, 4]. Delayed diagnosis often presents when the disease has already reached an advanced stage; these cancers also present symptoms frequently overlooked, including abnormal bleeding, non-healing ulcers and pelvic pain.

Evidence revealed increased age is itself a significant risk factor for vulvar and vaginal cancers. Other risk factors include smoking, early age of first sexual intercourse, history of multiple sexual partners (MSPs), history of sexually transmitted infections, low socioeconomic status, history of carcinoma, prior hysterectomy, immunosuppression, presence of genital warts, chronic inflammation or lichen sclerosis [1, 2]. The strongest risk factors for vulva and vaginal cancers are human papillomavirus infection, immunosuppression and advanced age [1, 5].

The most presenting symptoms of vulvar and vaginal cancer are localised pruritus, unusual bleeding, local pain, surface drainage from the tumour, vaginal discharge, blood in urine or stool, sexual intercourse pain, frequent urge to urinate, vulvar mass with endophytic or exophytic lesions ranging from a small vulvar swelling to massive vulvar masses ulcerations and urinary tract symptoms [2].

The staging of vulvar and vaginal are commonly conducted according to the International Federation of Gynecology and Obstetrics (FIGOs) system [1]. This staging method helps evaluate the extent of the disease and influences treatment decisions. The 5-year survival rates vary significantly dependent on the stage at diagnosis ranging from 86% for early-stage disease (FIGO stage I) to 19% for metastatic disease (FIGO stage IVB) and the lifetime risk of developing vulvar and vaginal cancer is approximately 0.3% [1].

The treatment of vulvar cancer depends on the disease’s histology, stage and patient’s performance status and includes surgical, chemotherapy, radiotherapy, palliative care or multimodality treatment process [5]. Surgery is the mainstay of treatment, which has evolved from extensive radical vulvectomy with significant postoperative complications to less radical surgery with better quality-of-life outcomes [6]. Furthermore, lymphadenectomy remains an integral part of radical vulvectomy, as lymph node positivity is a significant prognostic factor. Centrally localised vulvar tumours require bilateral lymph node dissection, usually performed using incisions in the groin. Common surgical methods for early-stage disease include hemivulvectomy, excision biopsy and wide local excision [6]. Unfortunately, in developing countries, many patients present with advanced-stage diseases attributed to factors such as social stigma, low socio-economic status and illiteracy [7].

For patients with advanced vulvar cancers, post-operative regional radiotherapy has been demonstrated to reduce the risk of regional recurrence and increase overall survival [8]. However, only radiotherapy may not be sufficient to eliminate gross residual disease and does not address distant microscopic metastases, which may occur in 11%–12% of patients at 6 years [8]. The rates of poor survival following regional failure emphasise on the need for more effective initial treatments [8]. In vaginal cancer cases, surgery might be recommended for patients with early-stage disease. However, it is usually not feasible for those with advanced stage disease due to the closeness of the vagina to critical organs such as the rectum, bladder, ureters and urethra [8]. As a result, the standard of care for these patients typically involves a combination of external beam radiotherapy and brachytherapy.

This study aims to evaluate the presentation and management of VVCs at the NSIA-LUTH Cancer Centre over a specified period. This study will also seek to identify risk factors and clinical features, staging classification, incidence, prevalence and treatment modalities that would enhance patient outcomes. This study is necessary to guide evidence-based policies for improved quality of cancer care in Nigeria and other similar settings.

## Methods

Out of 10,376 cancer patients from May 2019 to June 2024, 73 of them were pathologically proven and documented as VVCs as the shortest, but 6 were excluded due to incomplete staging or missing clinical details. The data of this study were obtained retrospectively from the NLCC Cancer patients’ database at the Department of Research, NSIA-LUTH Cancer Centre, Lagos, Nigeria. All patients were diagnosed with VVCs by tumour biopsy and/or radiological imaging. Inclusion criteria for VVCs included the availability of well-documented clinical information from the electronic medical record. Exclusion criteria for this study included patients with other concurrent malignancies.

Data were gathered from age at presentation, marital status, religion, ethnicity, menopausal status, presenting symptoms, comorbidities, smoking, alcohol, family history of cancer and staging. Furthermore, treatment modalities were obtained. All parameters were systematically entered into a computerised database for analysis. Ethical approval was not required for this study.

### Statistical analysis

The data were statistically analysed using the Statistical Package for Social Science version 27. The results obtained were subsequently presented in tables, relative frequencies and percentages.

## Results

Table 1 shows patients’ socio-demographic characteristics. The mean age at presentation was 54.69 ± 12.52 years (range 32–88). Out of 67 patients, 35.8% were within 41–50 years and 13.4% were above 71 years of age. More than half of the population (79.7%) were married in this study, single (14.9%) and widows (6%). Religion results revealed 84.9% were Christians and 6.8% were Muslims. The most common ethnic group was Yoruba (44.8%) and other tribes (38.8%), and the least common was Igbo (16.4%). Most of the patients with VVCs were post-menopausal (70.1%).

Table 2 demonstrated patients’ risk factors and clinical presentation of VVC patients. Clinical symptoms showed that vaginal bleeding and lump were the most common presenting symptoms with 41.8% each. Other clinical presentation were itching (29.9%), vaginal swelling (32.8%), vagina discharge (29.9%) and ulcer (22.4%). Comorbidities were present in 50.7%. Histological results revealed that the most common (74.9%) histological sub-type found in this study is squamous cell carcinoma. There were cases of adenocarcinoma (13.4%) and verrucous carcinoma (3.0%). However, other histological subtypes reported were Bartholin gland carcinoma, clear cell carcinoma, mucinous carcinoma, high-grade sarcoma, malignant spindle cell tumour and invasive melanoma, each accounted for 1.5% (1 patient). The most common risk factors for VVC were MSP (7.5%), family history of cancer (7.5%), immunosuppression (22.3%), smoking (1.5%) and alcoholics (25.4%).

Table 3 examined the FIGO staging classification of VVC patients. Fifty (72.6%) patients were presented in advanced stages of the disease (stages III and IV) and 17 (25.3%) patients were in early-stage disease (stages I and II).

Table 4 shows the incidence and prevalence of VVC patients. The incidence of VVC was 0.8%, 0.5%, 0.5%, 0.5% and 0.7% in 2020, 2021, 2022, 2023 and 2024, respectively. The prevalence of VVC in this study was 0.6%.

Table 5 revealed that the treatment modalities available to VVC patients were surgery (23.9%), chemotherapy (40.3%) and radiotherapy (56.7%).

## Discussion

The prevalence of VVC in this study was 0.6%, with 67 cases identified among the 10,376 total cancer cases recorded within the study period, and this finding was contrary to other studies done in other parts of Nigeria. Notably, there has been a downward and upward trend in the vulvovaginal incidence rate over the years. Persistent HPV infection, smoking, MSP, early age at first sexual intercourse, exposure to diethylstilbestrol (DES), sexually transmitted infections such as Herpes Simplex and Papilloma Virus, immunosuppression Human Immunodeficiency Virus (HIV), family history of cancer, personal history of cervical cancer and low socio-economic status [9] has been implicated in this trend. This study’s finding was lower compared to the US (27.8%), Senegal (2.7%), Cameroon (4%) and Gabon (2.21%) [9].

The mean age at presentation was 54.69 ± 12.52 years, varying from 32 to 88 years. Twenty-four patients (35.8%) in this study were within 41–50 years. The age at presentation in this study was contrary to other studies obtained in Nigeria and other developed countries [9–11]. Studies have revealed that VVC is a relatively rare cancer, which occurs mostly among older women [2, 9]. However, over the years, there has been a noticeable shift with an increasing occurrence of VVC among younger women [9]. The clinical symptoms obtained in the study were similar with Alkatout *et al* [12], Goody and Oko [2] and National Cancer Institute [13], who reported bleeding, discharge, lump, ulcer, dysuria and pain.

The prevalence of cancer comorbidities in this study was 50.7%, which could be a result of smoking and alcohol observed in this study. Comorbidities observed in this study were hypertension, diabetes and HIV. Comorbidities can affect the timing, delivery or outcomes of cancer treatment [14]. Studies have shown that patients with comorbidities are less likely to receive radical treatment, compared to those without comorbid conditions. However, there is increasing evidence that many patients with comorbidities can still benefit from such treatment [14]. Squamous cell carcinoma was the most common histological subtype observed in this study, which accounts for 74.8%. This was consistent with other studies reported in Nigeria [2, 15], sub-Saharan Africa [9] and developed countries [5, 12, 13, 16, 17]. Adenocarcinoma accounts for 13.4% of cases, and one case each of verrucous carcinoma, Bartholin carcinoma, clear cell carcinoma, mucinous carcinoma, sarcoma, spindle cell tumour and melanoma. Studies have reported that other histologic subtypes of VVC are rare [9, 12, 18, 19]. Clear cell carcinoma in this study could be associated with in-utero exposure to DES [15].

This study reported 7.5% of patients with MSP. This was consistent with other studies reports [2, 9]. MSP has been associated with an increased risk of VVC. Furthermore, MSP may result in the introduction of other sexually transmitted pathogens, such as HIV, which is known to increase the risk of VVC [9]. Family history was observed in 7.5% of patients, which indicated first- and second-degree relatives in this study. This may be due to inherited genetic mutations in tumour suppressor genes or oncogenes, such as BRCA1, BRCA2 or other related pathways [20]. Evidence has shown that VVC patients with a family history have a higher likelihood of sharing genetic or environmental factors contributing to cancer development.

Almost a quarter (22.3%) of the patients in this study were diagnosed with HIV. The findings of this study were low compared to the 37.5% reported by Goddy and Oko [2]. Evidence demonstrated that the presence of HIV-induced immunodeficiency plays an important role in promoting HPV infection and its persistence in HIV-positive patients [9]. Moreover, this immunodeficiency makes these patients more susceptible to developing genital cancer, including vaginal and vulva cancer [21].

The most common cases of VVC in this study were diagnosed at advanced stages of the disease (stages III and IV). The population of patients presented with stages III and IV cancer was 74.6%, which was low compared to 100% reported by Goddy and Oko [2]. This could be due to geographical location, low health illiteracy, lack of awareness, sociocultural beliefs, lack of financial resources, poor diagnostic procedures, poverty and limited treatment facilities [2, 9, 11]. This is alarming considering that external genitalia are easily accessible, but most patients will not seek help until the condition becomes unbearable [2]. Studies have shown that late presentation can lead to complicated diagnosis and treatment, poor prognosis and increased mortality rates [5].

Treatment modalities available for VVC in this study were surgery (23.9%), chemotherapy (40.3%) and radiotherapy (56.7%). Evidence has shown that due to the rarity of the disease and the absence of randomised trials, management of this aggressive disease is masked with dilemmas and controversies [7]. However, surgery has been known to be the cornerstone in the management of VVC, especially in the early stages, and associated comorbidities cannot be ignored. Furthermore, studies reported in India indicated that the surgery option is often replaced by chemotherapy and radiotherapy for vulvovaginal patients presented at an advanced stage of the cancer [7].

## Conclusion

This study reported a prevalence rate of 0.6% VVC, with a mean of 54.69 years and a shift toward younger women. Squamous cell carcinoma was the most common histological subtype observed with risk factors, such as immunosuppression, alcohol use, smoking, MSP and family history of cancer.

## List of abbreviations

DES, Diethylstilbestrol; FIGO, International Federation of Gynecology and Obstetrics; HIV, Human Immunodeficiency Virus; MSP, Multiple sexual partners; VVC, Vulvovaginal cancer.

## Statements and declarations

This study is an original work and is the fruit of various researchers contributing in various capacities.

## Conflicts of interest

The authors have no relevant financial or non-financial interests to disclose.

## Funding

No funds, grants or other financial support were received in the preparation of this manuscript.

## Ethical approval

This study obtained Ethical approval from the Health Research and Ethics Committee of the Lagos University Teaching Hospital, Lagos.

## Author contributions

All authors contributed to the study’s conception and design. Chidiebere I Agbakwuru and Olumide A Noah performed material preparation, data collection and analysis. Agbakwuru wrote the first draft of the manuscript and all other authors commented on previous versions. Everyone read and approved the final manuscript.

## Figures and Tables

**Table 1.** Socio-demographic characteristics of VVC patients.

Characteristics	Frequency	Percentage
**Age at presentation**		
°30–40 years	7	10.4
°41–50 years	24	35.8
°51–60 years °61–70 years °71 and above	14 13 9	20.9 19.4 13.4
**Marital status**		
°Married	53	79.7
°Widowed °Single	10 4	6.0 14.9
**Religion**		
°Christianity °Islam	62 5	84.9 6.8
**Ethnicity** °Yoruba °Igbo °Delta °Edo °Others	30 11 4 4 15	44.8 16.4 5.9 5.9 27.0
**Menopausal status**		
°Premenopausal	47	70.1
°Peri-menopausal	15	22.4
°Post-menopausal	5	7.5

**Table 2.** Risk factors and clinical presentation of VVC patients.

Variables	Frequency	Percentage
**Clinical presentation**		
**Itching**		
°Yes	20	29.9
°No	47	70.1
**Vaginal swelling**		
°Yes	22	32.8
°No	45	67.2
**Vaginal discharge**		
°Yes	20	29.9
°No	47	70.1
**Lump**		
°Yes	28	41.8
°No	39	58.2
**Ulcer**		
°Yes	15	22.4
°No	52	77.6
**Vaginal bleeding**		
°Yes	28	41.8
°No	39	58.2
**Comorbidities**		
°Yes	40	50.7
°No	33	49.3
**Histology**		
°Squamous cell carcinoma	50	74.8
°Adenocarcinoma	9	13.4
°Verrucous carcinoma	2	3.0
°Bartholin carcinoma	1	1.5
°Clear cell carcinoma	1	1.5
°Mucinous carcinoma	1	1.5
°Sarcoma	1	1.5
°Spindle cell tumor	1	1.5
°Melanoma	1	1.5
**Risk factors**		
°MSP	5	7.5
°Family history of cancer	5	7.5
°Immunosuppression (HIV)	15	22.3
°Smoking history	1	1.5
°Alcohol history	17	25.4

**Table 3.** FIGO staging classification of VVC patients.

Characteristics	Frequency	Percentage
**Stage**		
°I	9	13.4
°II	8	11.9
°III	15	22.4
°IV	35	52.2

**Table 4.** Incidence and prevalence of VVC patients.

Year	New cases	Population size	Incidence rate
2020 2021 2022 2023 2024	12 10 11 13 21	1,517 1,795 1,938 2,260 2,866	0.8% 0.5% 0.5% 0.5% 0.7%

**Table 5.** Treatment modalities in VVC patients.

Modalities	Frequency	Percentage
**Surgery** °Yes °No	16 51	23.9 76.1
**Chemotherapy** °Yes °No	27 40	40.3 59.7
**Radiotherapy** °Yes °No	38 29	56.7 43.3
